# Critical Roles of *N*^6^-Methyladenosine (m^6^A) in Cancer and Virus Infection

**DOI:** 10.3390/biom10071071

**Published:** 2020-07-17

**Authors:** Ken Asada, Amina Bolatkan, Ken Takasawa, Masaaki Komatsu, Syuzo Kaneko, Ryuji Hamamoto

**Affiliations:** 1 Cancer Translational Research Team, RIKEN Center for Advanced Intelligence Project, 1-4-1 Nihonbashi, Chuo-ku, Tokyo 103-0027, Japan; abolatka@ncc.go.jp (A.B.); ktakazaw@ncc.go.jp (K.T.); maskomat@ncc.go.jp (M.K.); 2 Division of Molecular Modification and Cancer Biology, National Cancer Center Research Institute, 5-1-1 Tsukiji, Chuo-ku, Tokyo 104-0045, Japan; sykaneko@ncc.go.jp

**Keywords:** RNA modification, epigenetics, methylation, cancer, RNA virus

## Abstract

Studies have shown that epigenetic abnormalities are involved in various diseases, including cancer. In particular, in order to realize precision medicine, the integrated analysis of genetics and epigenetics is considered to be important; detailed epigenetic analysis in the medical field has been becoming increasingly important. In the epigenetics analysis, DNA methylation and histone modification analyses have been actively studied for a long time, and many important findings were accumulated. On the other hand, recently, attention has also been focused on RNA modification in the field of epigenetics; now it is known that RNA modification is associated with various biological functions, such as regulation of gene expression. Among RNA modifications, functional analysis of *N*^6^-methyladenosine (m^6^A), the most abundant RNA modification found from humans to plants is actively progressing, and it has also been known that m^6^A abnormality is involved in cancer and other diseases. Importantly, recent studies have shown that m^6^A is related to viral infections. Considering the current world situation under threat of viral infections, it is important to deepen knowledge of RNA modification from the viewpoint of viral diseases. Hence, in this review, we have summarized the recent findings regarding the roles of RNA modifications in biological functions, cancer biology, and virus infection, particularly focusing on m^6^A in mRNA.

## 1. Introduction

The central dogma of biology has been described by Francis Crick as transmission of information from genes (DNA) to proteins via RNA [1], which involves the consecutive steps of transcription and translation. Initially, RNA was considered only a temporal messenger that conveys the genomic information for protein synthesis. Recent studies have shown that non-coding RNAs (ncRNAs), including microRNA (miRNAs), which are not stated in the central dogma, play important roles in several cellular processes, and that their dysregulation is related to many diseases [2,3,4]. ncRNAs and their binding protein complexes regulate transcription by modulating the chromatin structure [5]. In addition, post-transcriptional modifications of RNA, which have recently emerged as epigenetic or epitranscriptomic modifications, have also been shown to regulate multiple biological processes, including development. Similar to other epigenetic modifications, alterations in RNA modifications are associated with the onset and progression of diseases, including cancer [6,7,8]. Thus, it is important to elucidate the detailed molecular mechanisms of RNA modifications for new diagnostic and therapeutic methods for diseases.

m^6^A is the most abundant reversible modification on mRNA and typically enriched in the 3′ untranslated region (3′-UTR). The m^6^A peaks are enriched at the near stop codon, which more than 60% of m^6^A peaks were detected in the first quarter of the 3′-UTR. In contrast, m^6^A is found at low levels in the 5′-UTR and in the 5′ end of the coding sequence (CDS). The m^6^A peaks increase in proportion to the transcript length and at the end of CDS is higher methylated than at the beginning [9]. Dominissini et al. reported that an average of one to three m^6^A peaks per transcript (one peak per 2000 nucleotides or 1.7 peaks per gene) were found in mammalian cells [10]. Furthermore, m^6^A modification has been detected in mRNAs and ncRNAs [11]. In addition to m^6^A, other types of modifications, such as m^1^A, Ac^4^C, m^5^C, and m^7^G, were reported in mRNAs, ncRNAs, tRNAs, rRNAs, and miRNAs from humans, mice, and plants [12,13,14,15,16,17,18]. More specifically, there are more than 100 modifications known in tRNA; m^1^A, m^1^G, m^1^Ψ, I, m^1^I, m^2^_2_G, m^3^C, Ac^4^C, m^5^C, nm^5^U, f^5^C, ms^2^t^6^A, acp^3^U, Um, and so on, and the functions related to disease are reported [19,20,21]. Although RNA modifications of the ribose group have been reported [22], we have mainly focused on modifications of nitrogenous bases, especially m^6^A in mRNA, in this review.

We have also discussed the relationship between RNA modifications and virus infections, including severe acute respiratory syndrome coronavirus 2 (SARS-CoV-2) infection, which is a newly identified virus that caused the coronavirus disease 2019 (COVID-19) pandemic worldwide since December 2019.

## 2. Chemical Mechanisms of Adenosine Methylation

An accumulating body of evidence indicates that RNA modification occurs on all four bases [23]. A consensus sequence, known as RRACH motif (R = A or G, H = A, C, or U), has been reported for the m^6^A modification [6,24,25,26]; however, in vitro methylation activity with different RNA probes did not reveal any structural preferences for the RRACH motif [27]. Of note, other studies for structural insights with icSHAPE (in vivo click selective 2′-hydroxyl acylation analyzed by primer extension), will be discussed in Section 3.1 (m^6^A Alters RNA Folding). Moreover, mRNA modifications are distributed in a position-specific manner; for example, m^1^A occurs near translation start codons and first splice sites, whereas m^6^A enriches in long coding sequences and the 3′-UTR [9,10,16,17], which is a regulatory element for mRNA, where miRNA and other proteins bind, and these position specificities are possibly important for RNA function. For instance, the m^6^A modification on mRNA modulates gene expression levels, stability, splicing, polyadenylation, export, and translation [9,10,11,22,26,28], i.e., most of the cellular events. Therefore, the relationship between RNA modifications and disease is being actively investigated. We will discuss the roles of m^6^A modification in cancer in Section 5 (*N*^6^-methyladenosine in cancer).

**Figure 1:**
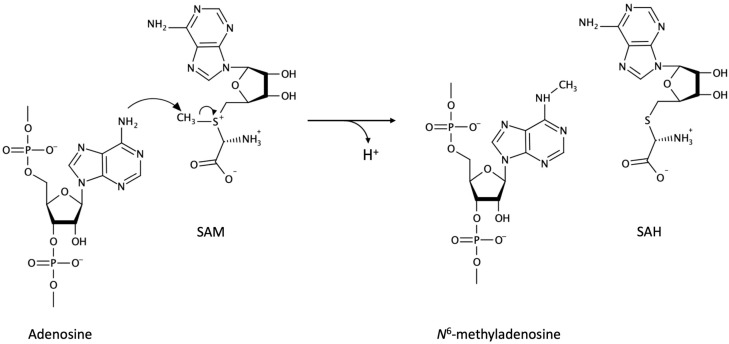
Molecular mechanism of *N*^6^-methyladenosine methylation. SAM: S-adenosylmethionine. SAH: S-adenosyl-L-homocysteine.

It is well-known that three components, the modification writer, reader, and eraser, are involved in RNA modification-dependent signaling. The first paper of m^6^A methyltransferase was reported in 1997, which indicates that methyltransferase like 3 (METTL3) is a key component of methylation complex [29]. Many important functions and mechanisms related the m^6^A modifications in RNAs were reported in the early 2010, as mentioned below. The writer protein METTL3 and METTL14 constitute the enzymatic core of a methyltransferase complex for m^6^A; the activity of which was reconstituted in vitro [27,28]. With regard to the molecular mechanism of m^6^A modification, the methyltransferase binds to the methyl donor S-adenosylmethionine (SAM) and RNA to generate m^6^A on the RNA, while SAM is converted to S-adenosyl-L-homocysteine (SAH) [30] (Figure 1).

Furthermore, other writer complex components were also identified. Wilms tumor 1-associated protein (WTAP) is a ubiquitously expressed nuclear protein that binds to METTL3 and METTL14, regulating nuclear speckle localization [31], and KIAA1429 or VIRMA in humans was identified as one of the 13 candidates associated with methyltransferase components in a proteomics analysis. The depletion of KIAA1429 in human A549 cells decreases m^6^A levels in mRNA [32]. The RNA-binding motif protein 15 (RBM15) and RBM15B interact with WTAP in HEK293T nuclear lysates and knockdown of RBM15 and RBM15B decreases m^6^A levels [33]. The zinc finger CCCH-type containing 13 (ZC3H13 or KIAA0853) forms a complex with WTAP, VIRMA, and RBM15, which regulates the complex localization in nucleus and promotes m^6^A modification on mRNA in flies [34]. Casitas-B-lineage lymphoma-transforming sequence-like protein 1 (CBLL1) or HAKAI, first reported as an E3 ubiquitin ligase using a combination of genetics, proteomics, and RNA biochemistry, was reported to be involved in the writing of the m^6^A modification in human and plants [35,36]. It is noteworthy that RBM15, RBM15B, ZC3H13, and CBLL1 were reported to be WTAP binding proteins to form a complex, implying the possibilities that other WTAP binding proteins might be associated with m^6^A methylation because WTAP was reported to bind to multiple proteins [37].

The YTH domain was identified as an RNA binding domain [38]. Biochemical purification following mass spectrometric (MS)-analysis revealed that the YTH domain family 1 (YTHDF1), 2 (YTHDF2), and 3 (YTHDF3) are primary members of m^6^A reader proteins, which devote to recognizing bases that undergo m^6^A methylation, participating in downstream translation, mRNA degradation, and accelerating the rate at which mRNA leaves the nucleus [10,26,39]. Humans contain two more YTH domain proteins, namely, YTHDC1 and YTHDC2. YTHDC1, preferentially binds m^6^A residues on *XIST* or other ncRNAs, such as *MALAT1* and *NEAT1.* In addition, experimentally tethered YTHDC1 and XIST rescue XIST-mediated silencing upon loss of m^6^A [33], while an RNA helicase containing YTHDC2 promotes the translation efficiency of mRNAs with m^6^A in coding regions [40]. Furthermore, YTHDC2 is strongly expressed in testis and is essential for regulating m^6^A transcripts to ensure meiotic gene expression required for male and female fertility, and spermatogenesis [41,42]. Additionally, insulin-like growth factor 2 mRNA-binding proteins 1–3 (IGF2BP1–3) have been recently identified as reader proteins. Unlike the decay-dependent pathways of YTHDF2, IGF2BPs enhance mRNA stability and translation [43].

**Figure 2:**
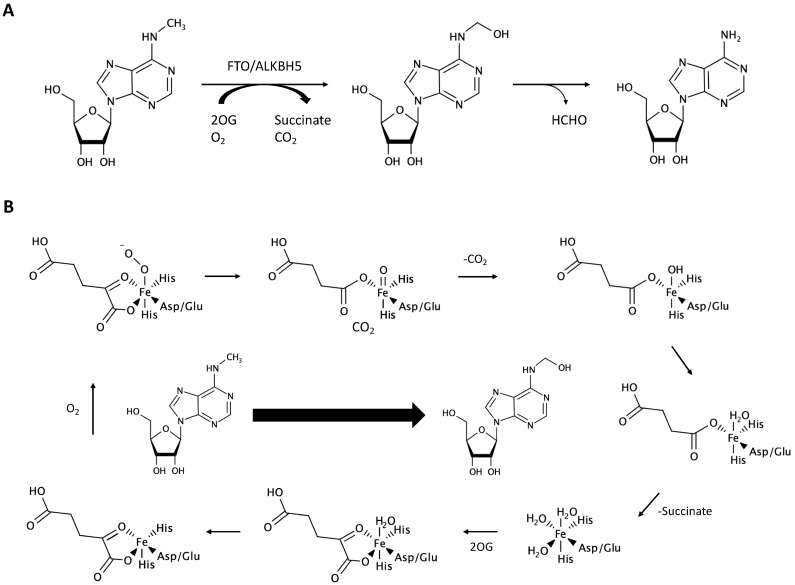
Molecular mechanism of *N*^6^-methyladenosine demethylation. (**A**) Overall biochemical process of m^6^A demethylation. (**B**) The enzymatic reaction of the first step of demethylation. FTO: fat mass and obesity-associated protein. ALKBH5: alkB homolog 5, RNA demethylase.

The last component is the eraser, which acts as a demethylase. Two demethylases have been well-investigated thus far; the fat mass and obesity-associated protein (FTO) and alkB homolog 5, RNA demethylase (ALKBH5). FTO removed modified m^6^A from RNA when recombinant human FTO was incubated with chemically synthesized m^6^A-containing single-stranded nucleic acids. In addition, siRNA-medicated knockdown of FTO decreased the expression levels of m^6^A by 23% in HeLa cells and by 42% in 293FT cells [44]. ALKBH5 overexpression and knockdown demonstrated that reduction in m^6^A levels in cells affected fertility. Importantly, ALKBH5 is mainly localized in nuclear speckles. Thus, ALKBH5 probably metabolizes m^6^A in nuclear RNA [45]. During the demethylation reaction, FTO or ALKBH5 catalyzes Fe (II)- and 2-oxoglutarate (2OG)-dependent demethylation with oxygen as an additional co-factor [46,47,48], as shown in Figure 2.

The detailed function of each of these components and their relationships to diseases will be discussed in Section 3 (biological functions of *N*^6^-methyladenosine) and 4 (*N*^6^-methyladenosine in cancer). The effect of m^6^A modification on virus infections will be discussed in Section 5 (significance of *N*^6^-methyladenosine for Virus Infection, including SARS-CoV-2).

## 3. Biological Functions of *N*^6^-Methyladenosine

**Figure 3:**
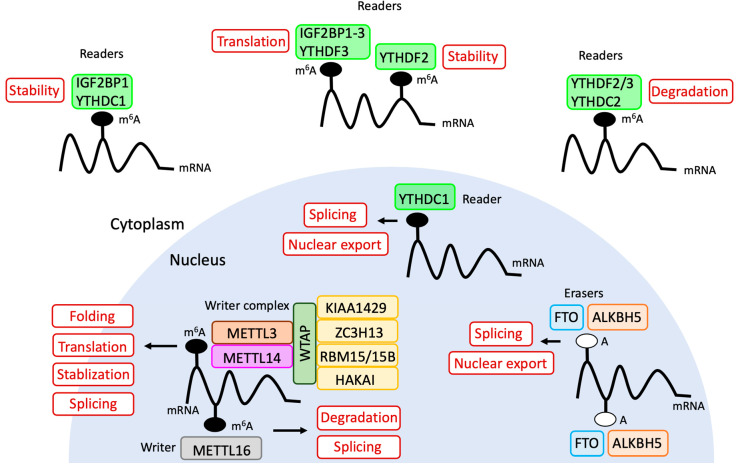
Biological functions of *N*^6^-methyladenosine. The main writer complex, readers, and erasers are involved in the processes of mRNA metabolism and mediate RNA folding, splicing, stability, transport, and translation.

The identification of the m^6^A modulators, as well as the advancements in next-generation high-throughput sequencing techniques, enabled determination of the biological functions of m^6^A in post-transcriptional processes and other diverse biological events. In particular, m^6^A is involved in the regulation of almost every aspect of mRNA metabolism, including, but not limited to, RNA folding, splicing, stability, transport, and translation (Figure 3).

### 3.1. m^6^A Alters RNA Folding

A recent study revealed that inclusion of m^6^A in three G:A base pairs abrogated RNA folding and ribosomal protein 7Ae 60S large ribosomal subunit (L7Ae) protein binding at the adenine A1n, and exerted a relatively mild effect at A2b and A3b positions, which may affect m^6^A recognition and its downstream biological regulation [49]. Another study showed that adenine may destabilize the duplex in the paired regions of RNA, leading to a less folded structure. In contrast, m^6^A stabilized the stretches of single-stranded RNA in unpaired positions as m^6^A stacked relatively stronger than the unmodified base [50]. Several research groups have identified a stronger RNA structural signature at m^6^A-modified sites than at unmodified positions, as well as significant loss of structural signals in *METTL3* knockout cells using a novel approach termed icSHAPE. Consistent with the results of other studies, these observations suggested that the destabilization effect of m^6^A on RNA helices enhances RNA structural signals [51].

### 3.2. m^6^A Affects RNA Splicing

Splicing of pre-mRNA is a dynamic and important process for gene expression. Intronic m^6^A has been linked to sex-lethal (Sxl) alternative pre-mRNA splicing, which specifically determines female sex [52]. Another study demonstrated that spliced exons and introns are enriched with m^6^A peaks, and silencing of METTL3 protein affects alternative splicing [10]. Liu et al. observed that m^6^A remodeled local RNA structure to enhance binding to heterogeneous nuclear ribonucleoprotein C (hnRNP C), a pre-mRNA splicing factor [53]. The other methyltransferase METTL16 is reported to play an important role in pre-mRNA splicing. METTL16, and the presence of its m^6^A substrate, a conserved hairpin (hp1) in the methionine adenosyltransferase 2 (*MAT2A*) 3′-UTR site, are necessary for the induction of *MAT2A* splicing, which promotes SAM synthetase expression [54]. Another report showed that METTL16-mediated m^6^A at position 43 of the U6 snRNA interacts with 5′ splice sites of pre-mRNAs during splicing [55,56]. The abovementioned studies showed that there is a specific adenine modification site underlined (UACAGAGAA) methylated by METTL16.

FTO and ALKBH5 also appear to regulate alternative splicing. FTO binds to pre-mRNAs in intronic regions, and *FTO* knockout leads to exon skipping events mediated by m^6^A [57]. Furthermore, FTO-dependent demethylation of m^6^A has been shown to control alternative splicing of Runt-related transcription factor 1 *(RUNX1T1*) by regulating the serine/arginine-rich splicing factor 2 (*SRSF2*) [58]. Notably, ALKBH5-mediated m^6^A is required for proper mRNA splicing, and the absence of ALKBH5 results in aberrant splicing of target genes, as well as decrease in serine/arginine-rich splicing factor 1 (SRSF1) signals [45,59].

The YTHDC1 has been proposed to affect mRNA splicing by recruiting the splicing factor 3 (*SRSF3*), which promotes inclusion of their targeted exons, while blocking serine/arginine-rich splicing factor 10 (*SRSF10*), which primarily facilitates exon skipping; this modification is dependent on the ability of YTHDC1 to recognize m^6^A regulation [60]. In addition, YTHDC1 interacts with SRSF3 and contributes to the regulation of mRNA splicing in oocytes in an m^6^A-based manner [61].

### 3.3. m^6^A Regulates RNA Stability

m^6^A is enriched in 3′-UTRs close to the stop codon, where RNA-binding proteins and other RNAs that modulate RNA stability are mostly localized. One of the best-established functions for m^6^A is to regulate mRNA stability. YTHDF2 is reported to destabilize the m^6^A containing mRNA, and YTHDF2 knockdown promotes the stability of target RNAs [26,62]. Another study showed that METTL3 knockdown, along with YTHDF2 deficiency, increases the RNA stability of the d2 isoform of vacuolar ATPase V0 domain (*ATP6V0D2*), and prolongs its half-life, indicating the impact of m^6^A in regulation of RNA stability [63]. METTL16 and YTHDC1 regulate the stability of the *MAT2A* mRNA via m^6^A methylation, which interacts with transcription factors and histone methyltransferases [56]. The impairment of the methyltransferase complex stabilizes the mRNAs of specific targets such as pluripotency factor Nanog homeobox (*NANOG*) upon the m^6^A deposition [64]. Interestingly, METTL14 knockdown increases the stability and translation of the MYC proto-oncogene (*MYC*) in an m^6^A-dependent manner [65]. m^6^A readers IGF2BPs have also been reported to promote the stability and expression of their target mRNAs, including *MYC,* by regulating m^6^A abundance [43]. Another study showed that depletion of FTO destabilizes its critical mRNA targets such as ankyrin repeat and SOCS box containing 2 (*ASB2*) and retinoic acid receptor alpha (*RARA*) due to reduction in m^6^A levels at these targets, which are important for differentiation of acute myeloid leukemia (AML) cells [66]. Previous studies have suggested that ALKBH5 is required for correct RNA splicing; in addition, ALKBH5-dependent m^6^A demethylation also regulates the stability of long 3′-UTR mRNAs in male germ cells [59].

### 3.4. m^6^A Mediates RNA Nuclear Export

Fustin et al. identified that specific inhibition of m^6^A methylation delays mRNA export, suggesting a role of the m^6^A modification in the mRNA export process [67]. Furthermore, ALKBH5 affects mRNA export due to its demethylation activity, and depletion of ALKBH5 accelerates nuclear to cytoplasmic export [45]. YTHDC1 has been demonstrated to modify mRNA splicing via interaction with splicing factors SRSF3 and SRSF10 [60]. Consistent with this role, YTHDC1 enhances nuclear export of m^6^A-modified mRNAs via the nuclear export pathway with SRSF3, the canonical mRNA export receptor, and nuclear transcription factor X-box binding 1 (NXF1), whereas YTHDC1 knockdown leads to deficient export of target mRNAs [68]. Interestingly, another study revealed that the m^6^A methyltransferase complex recruits the three prime repair exonuclease (TREX), a major mRNA export complex, to m^6^A-modified mRNAs, thereby driving efficient nuclear export [69].

### 3.5. m^6^A Modulates RNA Translation

Numerous studies have elucidated the important role of m^6^A in the regulation of mRNA translation. The reports showed that inhibition of m^6^A residues decreased the translation rate of dihydrofolate reductase by 20%, suggesting the presence of m^6^A in boosting translation efficiency [70]. YTHDF1 was found to elevate translation efficiency of m^6^A-modified mRNAs via interplay with translation initiation factor complex 3 (eIF3) and promote RNA loading onto ribosomes [71]. Furthermore, YTHDF2 promotes translation of structured mRNAs owing to the ability of m^6^A methylation to resolve mRNA secondary structures of CDS [40]. Studies have also shown that YTHDF3 enhances the translation of target mRNAs via interaction with YTHDF1 and YTHDF2 [72]. IGF2BPs, distinct m^6^A reader proteins, also enhance the translation of many target mRNAs in an m^6^A-dependent manner [43]. Interestingly, other studies indicated that METTL3 boosts the translation of m^6^A-modified mRNAs within the CDS by releasing ribosome stalling [73], and that METTL3 might directly facilitate translation of m^6^A-containing mRNAs near stop codons in cancer cells without the involvement of m^6^A reader proteins, including YTHDF1 or YTHDF2 [74]. Furthermore, m^6^A in the 5′-UTR promotes mRNA translation independent of initiation factor complex 4 (eIF4F), and ATP binding cassette subfamily F member 1 (ABCF1) plays a vital role in this process [75]. In contrast, Slobodin et al. demonstrated that enhanced m^6^A methylation in CDS impairs translation. In addition, m^6^A links transcription and translation via interaction with RNA polymerase II (RNAPII) and METTL3 [76]. One of the possible explanations is that m^6^A impacts translation depending on the location of the methylated residues either at UTRs or CDS.

### 3.6. m^6^A Regulates RNA Degradation

Studies have shown that proper m^6^A methylation is required for RNA degradation. A global increase in m^6^A levels induced by METTL3 enhanced degradation of mRNAs, including *NANOG*, SRY-box transcription factor 2 (*SOX2*), Kruppel like factor 4 (*KLF4*), and *MYC* [77]. Another study revealed that temperature stress re-localizes METTL3 and the DGCR8 microprocessor complex subunit (DGCR8) to stress-induced genes including heat shock protein 70 (Hsp70), for further degradation [78]. Studies have demonstrated that METTL16 and YTHDC1 regulate SAM-responsive RNA degradation of the *MAT2A* mRNA in the 3′-UTR via m^6^A modification [56]. Other than promoting mRNA translation, the YTHDF2-m^6^A-mRNA complex has been reported to control mRNA degradation [26], and YTHDF3 has been found to accelerate mRNA decay via cooperation with YTHDF2 [72]. YTHDC2 enhances the degradation of target mRNAs via interaction with a 5′-3′ exonuclease 1 (XRN1) in a m^6^A-dependent manner [41,42]. *ALKBH5* knockout increases in m^6^A levels and facilitates degradation of longer 3′-UTR transcripts during spermiogenesis, which also highlights the functional importance of m^6^A in mRNA degradation [59].

## 4. *N*^6^-Methyladenosine in Cancer

Emerging evidence suggests that m^6^A modification plays a critical role in various types of human cancers, including liver cancer, leukemia, lung cancer, breast cancer, glioblastoma, colon cancer, ovarian cancer, and other cancers. The molecular mechanisms underlying m^6^A-mediated regulation of proliferation, invasion, and migration of cancer cells, involve m^6^A writers, erasers, and readers.

### 4.1. m^6^A Writers in Cancer

The methyltransferase complex has been reported to have both a tumor-suppressor role and an oncogenic role in different cancers. Among the writers, METTL3 is widely studied and is known to be involved in various types of cancers. Recently, METTL3 was shown to be localized in the cytoplasm and nuclei of osteosarcoma cells, where it acted as an oncoprotein. METTL3 knockdown affected m^6^A methylation and impairs osteosarcoma cell proliferation and metastasis. Moreover, the ATPase family AAA domain-containing protein 2 (*ATAD2*), identified as the downstream target of METTL3, contributes to its oncogenic role in osteosarcoma [79]. METTL3 acts as an oncogene in colorectal cancer via its interaction with the glycolysis components, hexokinase 2 (*HK2*) and glucose transporter 1 (*GLUT1*) mRNAs, in an m^6^A-dependent manner. Studies have shown that METTL3 upregulates the m^6^A levels of *HK2* and *GLUT1*, depletion of which inhibits cancer cell proliferation and colony formation [80]. Similarly, the overexpression of METTL3 in colorectal cancer leads to abnormal m^6^A modification, and further promotes tumor progression and metastasis via its targets *SOX2* [81] and the METTL3/miR-1246/Sprouty-related EVH1 domain containing 2 (SPRED2) axis [82], respectively.

In bladder cancer, METTL3 is overexpressed, which correlates with poor prognosis. It has been suggested that METTL3 promotes bladder cancer cell proliferation and invasion by modulating pri-miR-221/222 [83] and the AF4/FMR2 family member 4 (AFF4)/nuclear factor kappa B subunit 1 (NF-κB)/MYC pathway in an m^6^A-dependent manner [84]. METTL3 is also an unfavorable prognostic factor in hepatocellular carcinoma, and knockdown of METTL3 suppressed hepatocellular carcinoma cell proliferation, colony formation, and migration in vitro. More importantly, suppressor of cytokine signaling 2 (*SOCS2*) was identified as a target of METTL3-mediated m^6^A modification in hepatocellular carcinoma [85]. METTL3 is associated with the m^6^A modified transcription factor CCAAT enhancer binding protein zeta (CEBPZ), and is required for the growth of AML cells [73]. Furthermore, m^6^A enhances the translation of *MYC*, BCL2 apoptosis regulator (*BCL2*) and phosphatase, and tensin homolog (*PTEN*) in AML, and METTL3 overexpression results in inhibition of leukemic cell differentiation [86].

In gastric cancer, METTL3-mediated m^6^A regulation plays an oncogenic role, and accelerates epithelial-mesenchymal transition (EMT) and metastasis via the METTL3/zinc finger MYM-type containing 1 (ZMYM1)/E-cadherin pathway [87], as well as the METTL13/heparin binding growth factor (HDGF)/glucose transporter 4 (GLUT4)/enolase 2 (ENO2) axis [88]. In pancreatic cancer, m^6^A and METTL3 are enriched in tumor specimens, and METTL3 boosts cancer cell proliferation, invasion, and migration via m^6^A modification [89]. Furthermore, depletion of METTL3 increases chemo- and radio-sensitivity in pancreatic cancer therapy [90]. It has been suggested that METTL3 plays an oncogenic role in lung cancer by regulating m^6^A containing mRNAs. METTL3 facilitates the translation of important oncogenes such as epidermal growth factor receptor (*EGFR*) and tafazzin (*TAZ*), a Hippo pathway effector, further regulating cancer cell growth, survival, and invasion [74]. In human endometrial cancer, researchers observed downregulation of m^6^A methylation in 70% in endometrial tumors, either because of the reduced METTL3 expression or METTL14 mutation. Notably, low levels of m^6^A in mRNA enhanced endometrial cancer cell proliferation and tumorigenicity via AKT serine/threonine kinase (AKT) signaling [91].

Interestingly, the role of m^6^A methylation in glioblastoma is conflicting. One study showed that m^6^A modification acts as a tumor suppressor by regulating glioblastoma stem cell growth, cell renewal, and tumorigenesis. METTL3 or METTL14 knockdown reduced m^6^A RNA levels, which elevated the expression of oncogenes such as EPH receptor 3 (*EPHA3*), *KLF4*, and ADAM metallopeptidase domain 19 (*ADAM19*), while it suppressed several tumor suppressor genes such as cyclin dependent kinase inhibitor 2A (*CDKN2A*), BRCA2 DNA repair associated (*BRCA2*), and tumor protein p53 inducible protein 11 (*TP53I11*) [92]. Nevertheless, the oncogenic role of METTL3 was shown in another study, where METTL3-mediated m^6^A modification enhanced *SOX2* mRNA stability, METTL3 knockdown decreased glioblastoma stem cell growth in a SOX2-dependent manner [93].

METTL14 was found to be highly expressed in AML, where it acted as an oncogene via m^6^A target genes such as MYB proto-oncogene transcription factor (*MYB*) and *MYC.* *METTL14* silencing leads to inhibition of AML cell growth and survival [65]. m^6^A levels and *METTL14* mRNA expression were low in renal cancer carcinoma, which were associated with poor overall survival. Mechanistically, METTL14-guided m^6^A modification suppresses the purinergic receptor P2X 6 (P2RX6) protein translation, which is important for renal cell carcinoma migration and invasion [94]. In hepatocellular carcinoma, METTL14 antagonizes METTL3 by suppressing tumor metastasis via interaction with DGCR8 [95]. Similarly, METTL14 inhibited colorectal cancer cell growth and metastasis by regulating its downstream target miR-375, which is a well-known tumor suppressor miRNA, revealing the role of METTL14-dependent m^6^A methylation in cancer [96].

WTAP has been reported as an oncogene in various cancers; for example, WTAP promotes metastasis of glioblastoma cells via EGF [97]. However, how WTAP affects human cancers in conjunction with m^6^A warrants further investigation. In hepatocellular carcinoma, WTAP has been shown to predict poor prognosis and acts as an oncogene. WTAP promotes hepatocellular carcinoma cell proliferation by silencing the expression of the tumor suppressor ETS proto-oncogene 1 (*ETS1*) transcriptional factor in an m^6^A-dependent manner [98].

### 4.2. m^6^A Erasers in Cancer

Increasing evidence has revealed that m^6^A eraser proteins regulate cancer-related biological processes. FTO has been reported to play a carcinogenic role in AML and promotes cancer cell proliferation and transformation by downregulating the m^6^A levels on several important cell differentiation related genes such as *ASB2* and *RARA* [66]. In lung squamous cell carcinoma, overexpression of FTO correlates with poor prognosis, as it dysregulates m^6^A levels. FTO enhances myeloid zinc finger protein 1 (*MZF1*) expression and further promotes cell proliferation and invasion by suppressing m^6^A levels [99]. ALKBH5, another important eraser of m^6^A methylation, has oncogenic roles in breast cancer [100] and glioblastoma [101], while it inhibits cancer cell migration and invasion in pancreatic cancer [102]. In breast cancer, overexpression of ALKBH5 promotes mRNA stability and expression of the pluripotency factor *NANOG*, which is required for primary tumor formation, and metastasis by catalyzing m^6^A demethylation [100]. In glioblastoma, increase in ALKBH5 expression is considered a poor prognostic factor. Forkhead box M1 (*FOXM1*), which is vital for glioblastoma stem cell proliferation and tumorigenesis, is significantly upregulated by ALKBH5 via its demethylation activity on m^6^A [101]. Interestingly, in pancreatic cancer, ALKBH5 is downregulated, which decreases cancer cell motility owing to demethylation of the m^6^A target lncRNA *KCNK15-AS1* [102].

### 4.3. m^6^A Readers in Cancer

m^6^A reader proteins, also known as binding proteins, includes YTHDF1-3, YTHDC1-2, and IGFBP1-3. m^6^A readers decide the ‘fate’ of m^6^A-modified mRNAs and play critical roles in cancer progression and tumorigenesis. YTHDF1 is overexpressed in colorectal cancer cells and acts as a poor prognostic factor. Studies have shown that of *YTHDF1* silencing inhibits cancer proliferation. More importantly, the *MYC* oncogene is related to the expression of YTHDF1 in this process [103]. YTHDC1 has been shown to interact strongly with the metadherin (MTDH) oncoprotein in prostate cancer, suggesting its role in cancer proliferation and tumorigenesis [104].

YTHDF2 acts as a tumor suppressor in cervical cancer. The lncRNA *GAS5-AS1* increases the expression of the tumor suppressor growth arrest specific 5 (GAS5) via the ALKBH5-m^6^A-YTHDF2 axis, regulating cancer growth and metastasis [105]. Moreover, YTHDF2 is upregulated in AML and is essential for cancer initiation and metastasis via regulation of m^6^A-modified transcripts [106]. Intriguingly, another study regarding the mechanism via which YTHDF2 affects AML showed that YTHDF2 binds to the *MYC* mRNA, which further enhances its stability and expression, antagonizing the action of the tumor suppressor R-2-hydroxyglutarate (R-2HG) in leukemic cells [107]. In hepatocellular carcinoma, YTHDF2 suppresses the mitogen-activated protein kinase 1 (ERK)/mitogen-activated protein kinase 7 (MEK) signaling pathway by reducing EGFR expression and subsequently inhibits cancer cell proliferation and growth [108]. YTHDC2 is overexpressed in human colon cancer and positively correlates with tumor metastasis via hypoxia-inducible factor 1a (HIF-1α) [109].

IGF2BP1 is upregulated in ovarian, skin, lung, and liver cancers, and enhances the expression of serum response factor (*SRF*) by elevating m^6^A modification, thereby accelerating cell proliferation and metastasis [110]. IGFBP2 is highly expressed in pancreatic cancer and is considered as an unfavorable prognostic factor. *DANCR*, a long non-coding RNA that enhances cancer cell proliferation and stem-like properties, is positively modulated by IGF2BP2 via m^6^A modification [111].

Taken together, we have comprehensively summarized recent studies regarding m^6^A modification and the molecular mechanisms of m^6^A modulators in regulating various human cancers, including tumor initiation, cancer cell proliferation, metastasis, and invasion (Table 1).

## 5. Significance of *N*^6^-Methyladenosine for Virus Infection, Including SARS-CoV-2

Similar to that observed in other species, recent studies have shown that virus RNA modifications play an important role in cellular events [115,116,117]. Studies have clearly demonstrated that m^6^A regulates human immunodeficiency virus (HIV) production, replication, translation, and reverse transcription, suggesting that the understanding of virus RNA modifications is as important as other areas of virology.

In this Section, we have summarized (1) the history of RNA modifications on viruses, (2) the relationship between m^6^A modification and viral infection, replication, and cellular immunity, and (3) m^6^A modification in SARS-CoV-2.

### 5.1. History of RNA Modification on Viruses

RNA modification of influenza virus was detected in 1976; kidney cells were infected with the virus in the presence of the radioactive material [118]. Subsequently, specific m^6^A methylation on Rous sarcoma virus RNA was observed after infection of the host cells. The virus RNA isolated from the host cells was hybridized with single-stranded phage DNA. After restriction enzyme digestion of nucleotides spanning approximately 6000 to 8000 bp, seven positive and four ambiguous m^6^A sites on the virus RNA were identified [119]. The biological function of m^6^A varies with different virus strains and host cells. To date, more than 10 viruses have been examined to determine the role of m^6^A after infection [120]. Furthermore, a recent study demonstrated that the demethylase activity of the eraser protein, ALKBH5, was impaired in host cells, which increased m^6^A expression on α-ketoglutarate dehydrogenase (*OGDH*) mRNA in response to viral infection, reducing mRNA stability and protein expression [121]. This indicated that the importance of RNA modification in the host cell metabolism.

### 5.2. The Relationship between m^6^A Methylation and Viral Infection, Replication, and Cellular Immunity

Many single-stranded RNA viruses (ssRNA) cause severe infectious diseases such as influenza, Ebola fever, Zika fever, severe acute respiratory syndrome (SARS), Middle East respiratory syndrome (MERS), and COVID-19. When viral genomic RNA is internalized and released into the cytoplasm after infection, the RNA is recognized by cellular RNA sensors such as stimulator of interferon response cGAMP interactor 1 (STING), Toll-like receptor 7 (TLR7), DExD/H-box helicase 58 (RIG-I), and others [122]. These sensors participate in the detoxification of the invading viruses via immune response, which is accompanied by the production of type I interferons (IFNs), inflammatory cytokines, and chemokines. However, RNA viruses are known to escape from these immune responses using various strategies.

*Flaviviridae* is a family of positive-sense ssRNA viruses represented by the Zika virus (ZIKV), dengue virus (DENV), and West Nile virus (WNV). The RNA genomes of several Flaviviridae viruses have been reported to contain m^6^A modification. For example, Gokhale et al. reported that the m^6^A in the genomic RNAs of ZIKA, DENV, WNV, yellow fever virus (YFV), and hepatitis C virus (HCV) can be detected by using methylated RNA immunoprecipitation sequencing (MeRIP-seq) [123], and Lichinchi et al. reported that ZIKV infection enhances m^6^A levels in the 5′-UTR region of cellular RNAs [115]. Despite the identification of these 51 genes, the alterations in m^6^A levels in the 5′-UTR region were not statistically significant [123]. Although we can argue regarding the qualitative versus quantitative nature of the data, this discrepancy should be addressed using biochemical assays, such as the site-specific cleavage and radioactive-labeling followed by ligation-assisted extraction and thin-layer chromatography (SCARLET) method [124], or a novel sequencing method [125], ideally coupled with functional assays for identifying the effect of infections on RNA modifications. It is noteworthy that these 51 genes are associated with antiviral and proviral functions, suggesting that further studies are required to better classify the function of m^6^A modification.

Similar to positive-sense ssRNA viruses, m^6^A modification in negative-sense ssRNA viruses has also been reported. For example, the RNA of HMPV (human metapneumovirus) harbors the m^6^A modification [126]. Therefore, increased expression levels of viral proteins were observed in A549 cells experimentally infected with HMPV, along with the overexpression of METTL3 and METTL14 and YTHDF1-3. Interestingly, infection with intact (m^6^A-unmethylated) HMPV activates the expression levels of RIG-I, followed by the activation of IFN-I and NF-κB pathways [126]. Furthermore, another study showed that IFN-β is significantly activated in cotton rats infected by intact HMPV, showing low virulence and significant induction of virus-neutralizing antibodies at two weeks post-infection [126]. Importantly, these results suggested that m^6^A methylation in HPMV is critical for escaping RIG-I-mediated host immune mechanism, and that intact HMPV may be useful as an attenuated vaccine [126].

### 5.3. m^6^A Methylation in SARS-CoV-2

As discussed previously, many RNA viruses contain the m^6^A modification. Here, we have focused on RNA modifications of the severe acute respiratory syndrome coronavirus 2 (SARS-CoV-2), which is a positive-sense, single-stranded RNA virus that causes a potentially lethal COVID-19 respiratory tract infection. As COVID-19 is now one of the most life-threatening diseases worldwide, many researchers are actively investigating SARS-CoV-2 strains to combat this disease. The basic background of SARS-CoV-2 has been summarized by a Turkish group [127]. To tackle this global pandemic, a deeper understanding of the mechanisms underlying SARS-CoV-2 infection, replication, or RNA modifications, which may contribute to the development of vaccines or drugs, is urgently required.

Partial sequences of coronaviruses (mouse hepatitis viruses) have been previously reported [128,129,130]. A recent study regarding SARS-CoV-2 demonstrates two important findings [131]. First, SARS-CoV-2 expresses genomic RNA, the sub-genomic RNAs of which consist of nine elements. Second, total RNA extracted from Vero cells with or without SARS-CoV-2 infection was sequenced using Nanopore direct RNA sequencing. This study revealed the SARS-CoV-2 transcriptome and epitranscriptome map, which allowed us to visualize RNA modification sites. The authors excluded METTL3-mediated m^6^A modification owing to the absence of a consensus RRACH motif, but observed at least 41 RNA modification sites, of which AAGAA and AAGAA-like A/G-rich motifs were prominently modified compared to the unmodified controls. In another cohort study in China, involving patients admitted from January 17th to February 8th 2020 [132], the authors detected clinically important features in the patients, as well as novel m^6^A modification loci in the Spike (S) protein, which are the m^6^A modification sites of SARS-CoV-1 and SARS-CoV-2 (Wuhan-Hu-1, ZJ01) using a bioinformatics approach. The importance of S proteins for SARS-CoV has been already discussed elsewhere [133]. Viral S proteins bind with angiotensin I converting enzyme 2 (ACE2) to get entered into the host cells. Identification of the potential m^6^A sites for S proteins in SARS-CoV-1 and SARS-CoV-2 indicated that m^6^A sites differ among viruses. Although the size of the genomic RNA size varies, flaviviruses, such as ZIKV and DENV, are estimated to possess 5–12 m^6^A modifications [115,123], while HIV-1 has 10–14, HCV has approximately 16, and influenza A virus contains up to 24 sites [116,118,134,135].

These results indicate that virus RNA modification is common and, hence, further studies are required for a comprehensive understanding of the relationship between RNA virus, infection, RNA epigenetics, and host cell immunity.

## 6. Concluding Remarks

The diverse biological roles of epigenetics and RNA modifications or epitranscriptomics have been elucidated, and epigenetic aberrations have been associated with various types of diseases, including cancer [136,137,138,139,140,141,142,143,144,145,146,147,148]. Focusing on RNA modifications, studies of m^6^A are a new boundary of disease, as we reviewed in this paper. This new additional layer of epigenetics facilitates an understanding of novel insights underlying cancer development, metastasis, drug response, and immune response induced by virus infection. m^6^A modifications are enriched in mRNA, especially in the 3′-UTR, but the diverse modifications are reported in other functional RNAs such as tRNA and rRNA, which are also associated with disease.

The importance of comprehensive understanding of RNA modifications in virus has surged these days due to COVID-19 pandemic worldwide. The WHO raised the threat of this epidemic to the “very high” level on February 28th, 2020. The SARS-CoV-2 genome, which is 30 kb in length, encodes a large non-structural protein, which is further cleaved to generate nine elements (four structural and five accessory proteins), as we discussed in Section 5.3. One of the four structural proteins is the S surface glycoprotein. The S protein sequence is believed to be methylated and assuming to affect the virus infection and virus replication. Thus, further active research is strongly desired.

Notably, the development of therapeutic agents targeting epigenetics is also progressing rapidly [149,150,151]. Indeed, multiple chemical inhibitors targeting RNA modification and RNA-editing enzymes are now known [47,152,153], and the estimated phase I trial will start in 2021 [152]. As discussed in the previous sections, m^6^A modification on RNA influences many cellular events; this cumulative knowledge can be expanded to investigate cancer, virus infection, and host innate immunity. Overall, detailed insight regarding RNA epigenetics or epitranscriptomics will assist in developing safe and effective drugs for cancer, COVID-19, and other diseases.

## Figures and Tables

**Table 1:** The role and mechanisms of m^6^A modulators in human cancer.

m^6^A Modulators	Cancer Types	Roles	Mechanisms	Ref.
**METTL3**	Osteosarcoma	Oncogenic functions	m^6^A methylation level and METTL3 expression are both upregulated in osteosarcoma tissues and cell lines. Promotes cancer cell proliferation and metastasis by regulating ATAD2	[79,112]
	Colorectal cancer (CRC)	Oncogenic functions	METTL3 can stabilize *HK2* and *GLUT1* expression in CRC through an m^6^A-IGF2BP2/3-dependent mechanism.	[80]
			METTL3 maintains *SOX2* expression through an m^6^A-IGF2BP2-dependent mechanism in colorectal cancer cells, and can work as a potential biomarker panel for prognostic prediction in CRC.	[81]
			METTL3/miR-1246/SPRED2 axis plays an important role in tumor metastasis.	[82]
	Bladder cancer	Oncogenic functions	METTL3, significantly increased in bladder cancer, is correlated with poor prognosis of bladder cancer patients, and may have an oncogenic role in bladder cancer through positively modulating the pri-miR-221/222 process in an m^6^A-dependent manner.	[83]
			METTL3-mediated m^6^A modification promotes bladder cancer progression through AFF4/NF-κB/MYC signaling network.	[84]
	Hepatocellular carcinoma (HCC)	Oncogenic functions	METTL3 is frequently upregulated in human HCC and contributes to HCC progression through repressing SOCS2 expression in an m^6^A-YTHDF2-dependent mechanism.	[85]
	Acute myeloid leukemia (AML)	Oncogenic functions	Promoter-bound METTL3 induces m^6^A modification within the coding region of the associated mRNA transcript, and enhances its translation, which is necessary for the maintenance of the leukemic State.	[73]
			METTL3 is frequently upregulated in human AML, and controls expression of *c-MYC*, *BCL-2*, and *PTEN* in an m^6^A-dependent manner.	[86]
	Gastric cancer	Oncogenic functions	METTL3, overexpressed in gastric cancer, is correlated with poor prognosis of gastric cancer, and required for the epithelial-mesenchymal transition (EMT) process in vitro and for metastasis in vivo.	[87]
			Elevated METTL3 expression can promote tumor angiogenesis and glycolysis in gastric cancer through m^6^A modification of *HDGF* mRNA.	[88]
	Pancreatic cancer	Oncogenic functions	METTL3 is enriched in human pancreatic cancer, and can promote cell proliferation and invasion of pancreatic cancer cells.	[89]
			METTL3 can promote the chemoresistance and radioresistance of pancreatic cancer cells through regulation of several critical pathways, including MAPK cascades, ubiquitin-dependent process, and RNA splicing.	[90]
	Lung cancer	Oncogenic functions	METTL3 is upregulated in human lung adenocarcinoma, and can promote cell proliferation, survival, and invasion of human lung cancer cells through enhancing translation of certain mRNAs, including *EGFR* and *TAZ*.	[74]
	Endometrial cancer	Tumor suppressive functions	About 70% of endometrial tumors show reduced total m^6^A mRNA methylation, which is mediated by either decreased METTL3 expression or METTL14 loss-of-function mutation, and reduced m^6^A methylation could promote cancer cell growth through activation of the AKT pathway.	[91]
	Glioblastoma	Tumor suppressive functions	Knockdown of METTL3 or METTL14 induces changes in mRNA m^6^A enrichment, and enhances cell proliferation of glioblastoma stem cells (GSCs) through altering expression of several oncogenes and tumor suppressors, such as *ADAM19* and *CDKN2A*.	[92]
		Oncogenic functions	METTL3, upregulated in GSCs, is essential for GSC maintenance, and stabilizes *SOX2* mRNA in an m^6^A-dependent manner.	[93]
**METTL14**	Acute myeloid leukemia (AML)	Oncogenic functions	METTL14 is required for development and maintenance of AML through regulating its mRNA targets, including *MYB* and *MYC* in an m^6^A-dependent manner.	[65]
	Renal cancer carcinoma (RCC)	Tumor suppressive functions	METTL14 is downregulated in RCC tissues, and could abrogate P2RX6 protein level in an m^6^A-dependent manner.	[94]
	Hepatocellular carcinoma (HCC)	Tumor suppressive functions	METTL14, downregulated in HCC, is associated with metastasis through modulating the processing of miR-126 in an m^6^A-dependent manner, and works as a prognostic factor in HCC.	[95]
**METTL16**	Colorectal cancer (CRC)	Association with worse OS in rectal adenocarcinoma	METTL16 is abundantly expressed in colon adenocarcinoma, and associated with the clinical outcomes of CRC patients.	[113]
		Mutational ITH and frameshift mutations with MSI-H	METTL16 harbors mutational intratumor heterogeneity (ITH) as well as the frameshift mutations in CRC with high microsatellite instability (MSI-H).	[114]
**WTAP**	Hepatocellular carcinoma (HCC)	Oncogenic functions	WTAP, highly expressed in HCC, is correlated with poor prognosis of HCC patients, and can promote cell proliferation of HCC cells through suppression of *ETS1* in an m^6^A-dependent manner.	[98]
**FTO**	Acute myeloid leukemia (AML)	Oncogenic functions	FTO is highly expressed in AMLs, and can enhance oncogene-mediated cell transformation and leukemogenesis through regulating its mRNA targets such as *ASB2* and *RARA* in an m^6^A-dependent manner.	[66]
	Lung squamous cell carcinoma (LUSC)	Oncogenic functions	FTO is a prognostic factor for LUSC, and can facilitate tumor progression in LUSC through regulating *MZF1* expression in an m^6^A-dependent manner.	[99]
**ALKBH5**	Breast cancer	HIF-depended enrichment of breast cancer stem cells (BCSCs)	Increased *NANOG* mRNA expression is induced by hypoxia in an HIF- and ALKBH5-dependent manner, which increased specification of BCSCs.	[100]
	Glioblastoma	Maintaining tumorigenicity of GSCs	ALKBH5 is highly expression in GSCs, and maintains tumorigenicity through regulating *FOXM1* expression in an m^6^A-dependent manner.	[101]
	Pancreatic cancer	Tumor suppressive functions	ALKBH5 inhibits pancreatic cancer motility through regulating lncRNA *KCNK15-AS1* expression in an m^6^A-dependent manner.	[102]
**YTHDF1**	Colorectal cancer (CRC)	Oncogenic functions	YTHDF1, highly expressed in CRC, is correlated with poor prognosis of CRC patients, and can promote cell proliferation of CRC cells.	[103]
**YTHDF2**	Cervical cancer	Tumor suppressive functions	lncRNA *GAS5-AS1* upregulates GAS5, a tumor suppressor, through an YTHDF2-dependent mechanism.	[105]
	Acute myeloid leukemia (AML)	Oncogenic functions	YTHDF2, overexpressed in human AML, is required for disease initiation, and decreases the half-life of diverse m^6^A transcripts that contribute to the overall integrity of self-renewing leukemic stem cell (LSC) function, including *TNFR2*.	[106]
	Hepatocellular carcinoma (HCC)	Tumor suppressive functions	Hypoxia induces downregulation of YTHDF2 in HCC cells, and YTHDF2 overexpression suppresses cell proliferation of HCC cells through inactivation of MEK and ERK.	[108]
**YTHDC1**	Prostate cancer	Potential tumor biomarker	The oncogene MTDH interacts with YTHDC1, KHDRBS1, and KHDRBS3, and modulates alternative splicing.	[104]
**YTHDC2**	Colon cancer	Promoting cancer metastasis	YTHDC2 can promote cancer metastasis through promoting HIF-1α.	[109]
**IGF2BP1**	Ovarian, skin, lung, liver cancer	Oncogenic functions	IGF2BP1 can promote the transcriptional regulator SRF-dependent transcription in an m^6^A-dependent manner.	[110]
**IGF2BP2**	Pancreatic cancer	Oncogenic functions	IGF2BP2, highly expressed in pancreatic cancers, is correlated with poor prognosis of pancreatic cancer patients, and can promote cell proliferation of pancreatic cancer cells through *DANCR*.	[111]
